# Cancer survivor rehabilitation and recovery: Protocol for the Veterans Cancer Rehabilitation Study (Vet-CaRes)

**DOI:** 10.1186/1472-6963-13-93

**Published:** 2013-03-11

**Authors:** Aanand D Naik, Lindsey A Martin, Michele Karel, Jennifer Schuster Wachen, Elizabeth Mulligan, Jeffrey S Gosian, Levi Ian Herman, Jennifer Moye

**Affiliations:** 1 Houston VA Health Services Research and Development Center of Excellence, Michael E. DeBakey VA Medical Center, Houston, TX, USA; 2 Section of Health Services Research, Department of Medicine, Baylor College of Medicine, Houston, TX, USA; 3 VA Boston Healthcare System, Boston, MA, 02130, USA; 4 Harvard Medical School, Boston, MA, 02115, USA; 5 Boston University School of Medicine, Boston, MA, 02118, USA

**Keywords:** Cancer care, Cancer survivorship, Veterans, Aging, Psychosocial, Functional assessment, Quality of life

## Abstract

**Background:**

Cancer survivors are a rapidly growing and aging population in the U.S., but there are many challenges associated with the survivorship experience such as functional disabilities and psychosocial distress. When viewed next to the general population, Veterans are especially at risk for these challenges as they are older and have a high incidence of co-morbid conditions. While the Institute of Medicine (IOM) has called for further cancer survivorship research to address these challenges, we still know little about this experience from the perspective of aging Veterans.

**Methods/design:**

We conducted a longitudinal, mixed-methods study over the course of three and a half years at the Boston and Houston VA Medical Centers. We recruited 170 Veterans diagnosed with head and neck, colorectal and esophageal/gastric cancers that were identified from the VA tumor registry. Veterans completed three in-depth interviews, conducted at 6, 12 and 18 months after pathology confirmation, measuring the physical, social and psychological factors related to cancer survivorship. The longitudinal design allowed us to assess any changes in cancer related disability and distress over time.

**Discussion:**

Weekly teleconference study team meetings were a key aspect to the research process. Issues related to recruitment, data management and analysis, and the dissemination of research results was discussed. Interviewers presented detailed case reports of completed interviews that allowed us to refine our interview protocols. We also discussed issues relevant to the Veteran population of which we were previously unaware and some of the challenges of the research process itself. This novel study produced a robust data set that documents the functional and psychosocial cancer survivorship experiences of aging Veterans. The longitudinal design will help us more fully understand the recovery patterns for this specific population, and identify the unique needs and gaps in health services.

## Background

 Cancer survivorship has steadily increased in prevalence since the mid-1970s [1-3] and currently there are over 13 million survivors in the United States alone [3,4]. The number of cancer survivors – defined as “any person who has been diagnosed with cancer, from the time of diagnosis through the balance of life” [2] – is likely to increase by 5 million over the next 10 years [3]. Cancer survivors are a rapidly growing and aging population [3], but the survivorship experience is frequently a double-edged sword. Pain, psychosocial distress and the onset of functional disabilities are long-term costs that cancer survivors often must face as they move forward with their lives [5].

Cancer type and stage, psychosocial stresses and supports, co-morbidities, and diverse treatment protocols make survivorship a variable experience [6,7]. Adults age 40 and over comprise the majority of cancer survivors in the U.S. today; the greatest proportion of this population is over the age of 65 [3,5]. Consequently, age is one such factor that complicates survivorship. The long-term effects of cancer treatment can hasten and/or worsen chronic co-morbid conditions (e.g. diabetes) and functional declines that are part of the normal aging process [8-13]. Viewed next to the U.S. population, Veterans of the U.S. military are at an especially high risk for these consequences as they are typically older, have higher rates of co-morbid conditions and may also have combat related post-traumatic stress disorder (PTSD), all factors complicating their survivorship experience [14-17].

The Institute of Medicine’s (IOM) influential report *From Cancer Patient to Cancer Survivor: Lost in Transition*[5] draws attention to these long-term physical and psychosocial effects of cancer treatment, and calls for research to improve and implement appropriate survivorship care. Survivorship research has grown over the years in response to the IOM’s call [18], yet we still know very little about cancer survivorship from the perspective of aging Veterans [17]. Therefore, the goal of this study is to recognize the functional and psychosocial effects of cancer diagnosis and treatment from diagnosis to 18 months for aging Veterans diagnosed with head and neck, colorectal, and esophageal/gastric cancers, and to identify the unique survivorship needs of this population. Results from this study will help us more fully understand the recovery patterns for aging Veteran cancer survivors and identify potential areas for survivorship interventions.

### Specific aims & hypotheses

#### Aim 1 & hypotheses

 To determine the longitudinal course of functional disability and psychological distress. We hypothesized significant distress and disability at 6 months, with improvement in a portion over 18 months, will be predicted by disease stage and treatments, age, social support, and mental health history.

#### Aim 2 & hypotheses

 To determine the impact of disability and distress on community integration. We hypothesized reintegration to community roles will be predicted by distress and functional disability, as moderated by the use of rehabilitative interventions and reports of post-traumatic growth.

#### Aim 3 & hypotheses

To characterize gaps in Veterans Health Administration (VHA) rehabilitative services for Veterans following cancer. We hypothesized high levels of overall satisfaction with Veterans Affairs (VA) medical care, with reports of lower satisfaction in emotional support and education, and moderate obstacles to receiving rehabilitative care. Further, we hypothesized moderate interest in receiving interventions (e.g. physical rehabilitation, mental health, complementary medicine and educational services), as moderated by levels of distress and disability.

#### Aim 4 & hypotheses

To characterize patient-centered goals for rehabilitation, community integration, and cancer survivorship treatment planning for Veterans following cancer. We hypothesized that this study will reveal previously unknown rehabilitation, community integration and cancer survivorship needs among this Veteran population.

## Methods/design

### Design overview

 To address these aims, we conducted a longitudinal, mixed methods study over a three and a half year period (November 2009-April 2013). Veterans completed in-depth interviews comprised of closed-ended survey responses and open-ended short answer questions that measured a wide variety of physical, social and psychological factors related to cancer survivorship. These interviews took place 6 (Time 1), 12 (Time 2) and 18 (Time 3) months (+/- 4 weeks for each time point) after pathology analysis confirmed malignancy. We chose to conduct three interviews in order to measure any change in cancer related functional disability and distress that occurred during the study period. Interviews were conducted in-person, or in rare cases, over the phone (n = 7) and lasted approximately two hours each. Veterans received compensation for their time after the completion of each interview ($30 per interview). This study received approval in August 2009 from the Boston VA and the Houston VA Institutional Review Boards (IRB) (Houston IRB# 25446; Boston IRB# 2317). Veterans completed a written consent form prior to beginning the study, which was entered into the VA Computerized Patient Records System (CPRS) and became part of the patient’s permanent medical records.

#### Screening & recruitment

**Figure 1:**
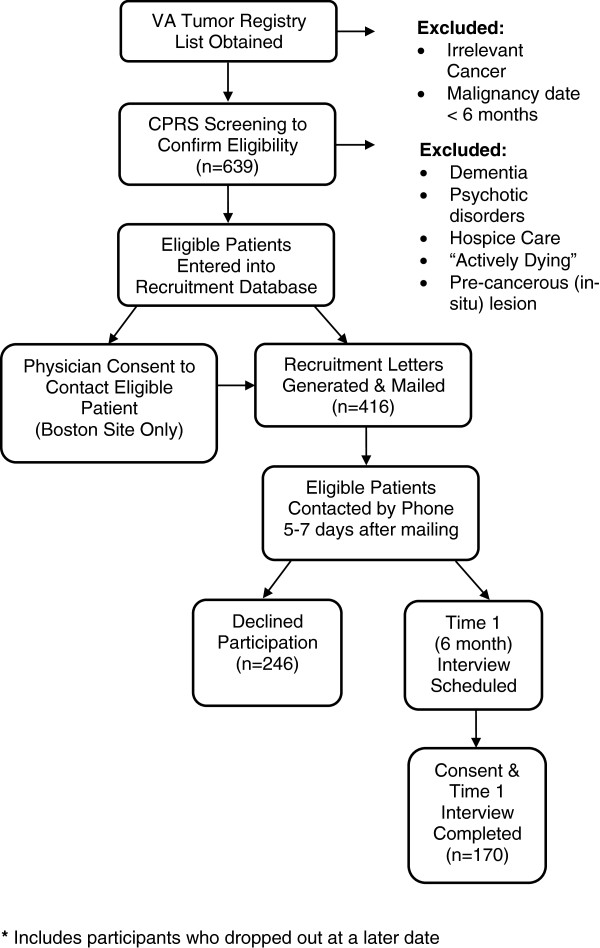
**Screening & recruitment process. **Overview of study screening and recruitment process arriving at Time 1 (6 month) sample.

After receiving a Health Insurance Portability and Accountability Act (HIPAA) waiver, we obtained lists of patients from the VA tumor registry whose diagnosis fell one month prior to the study’s opening eligibility window (6 months) (See Figure 1: Screening & Recruitment Process). Patients were eligible to be interviewed if their diagnosis fell within this window, and had a diagnosis of head and neck, esophageal, gastric, or colorectal cancer (see Additional file 1: International Statistical Classification of Diseases and Related Health Problems (ICD-9) Codes for Cancers included in the study). We utilized CPRS to screen patients to confirm diagnosis and to verify that the patient had been informed by their primary care physician or specialist of their diagnosis. We excluded patients who lacked the cognitive ability to be interviewed (patients diagnosed with Dementia or Psychotic Disorders on the basis of a medical record review), and patients receiving hospice care, considered “actively dying” or with a diagnosis of a pre-cancerous (in-situ) lesion. Eligible patients were entered into the study’s recruitment database. Prior to contacting eligible patients, our Boston site was required to obtain permission from the patient’s primary care physician or oncologist.

 Eligible patients were initially contacted via mail with a letter inviting them to participate in this study. This letter described the study’s purpose to learn more about Veterans’ cancer diagnostic and treatment experiences, explained that participation was completely voluntary and confidential, and that choosing to participate will not impact any present or future health care the Veterans receive through the VA. This letter also outlined the $30 compensation for the Time 1 interview, the interview location as well as the length of time for the interview. Eligible patients were informed that if they chose to participate, all study data was confidential and no identifying information would be revealed. A phone number was provided for further information. Five to seven days post-mailing, eligible patients were phoned by study coordinators. The purpose of this phone call was to confirm interest to participate, to provide further project information, to answer any questions the Veterans may have, and to schedule a time with the Veteran for written consent and the Time 1 interview.

 Veterans were contacted again at Time 2 (12 months) and Time 3 (18 months) with follow-up reminder letters informing them that study coordinators will be in contact to schedule or confirm their interview. Letters also informed the Veterans that they will be asked some of the same questions from the Time 1 interview, and some new questions. Also, Veterans were reminded that they would receive compensation for completion of each of these subsequent interviews ($30 each), and were provided a phone number to call if they had any questions.

 To account for Veterans who passed away or opted out of the study after completion of their Time 1 (6 month) interview, recruitment procedures were amended after the study began to enroll replacement patients on an as needed basis. These replacement subjects (n = 2) were expected to complete a Time 2 (12 month) and Time 3 (18 month) interview and received compensation accordingly ($30 per interview). The Time 2 interview (12 month) utilized many of the same survey instruments as the Time 1 interview, as well as collected a significant amount of new data providing study investigators the rationale to recruit these replacements. Replacements were drawn from those contacted for a Time 1 interview, but were unable to participate at 6 months due to the severity of their illness or if confirmation of their malignancy exceeded the initial 24 week criterion. New patients were also recruited at 12 months and underwent the same screening process outlined above.

#### Participants

A total of 170 Veterans were recruited for the baseline sample of our study (see Table 1: Demographic Characteristics of Baseline Time 1 Sample). Just over half of our sample (53.5%) was younger than age 65 while 46.5% was age 65 and older. The majority of this sample was male (98.2%) and almost 20% non-white. Nearly half (49.4%) had a high school education or less and 50.6% had attended college or earned a graduate degree. Most Veterans were diagnosed with either head and neck (40.0%) or colorectal (49.4%) cancers, with a smaller proportion diagnosed with esophageal/gastric cancers (10.6%). Using the American Joint Committee on Cancer’s (AJCC) tumor staging classifications, 23.7% of our sample was diagnosed at Stage I, 28.4% at Stage II, 21.9% at Stage III, and 26% at Stage IV. Nearly three quarters of our baseline sample (72.9%) had surgery and over half were treated with chemotherapy (60.6%) with fewer having received radiation therapy (41.1%). Most participants received a combination of two or more therapies.

**Table 1:** Demographic characteristics of baseline time 1 sample

**Characteristic**		**N = 170 (%)**
Age		
	<65	91 (53.5)
	65-74	50 (30.0)
	75-84	24 (14.1)
	85+	4 (2.4)
Sex		
	Male	165 (98.2)
	Female	3 (1.8)
Race/Ethnicity		
	White	137 (81.2)
	Non-white	32 (18.8)
Education		
	High school or less	83 (49.4)
	College/Graduate degree	86 (50.6)
Cancer Type		
	Head and neck	68 (40.0)
	Colorectal	84 (49.4)
	Esophageal/Gastric	17 (10.6)
Tumor stage		
	1	40 (23.7)
	2	48 (28.4)
	3	37 (21.9)
	4	44 (26)
Treatment received*		
	Surgery	124 (72.9)
	Chemotherapy	102 (60.6)
	Radiation	71 (41.1)

*Most participants received more than one treatment type.

## Methods

To measure longitudinal changes in Veterans functional disability and distress, the study’s three interview protocols utilized a diverse array of both quantitative and qualitative measures (see Table 2: Overview of Key Interview Measures). We selected these measures with both standardized and novel elements to provide a comprehensive assessment of the physical and psychosocial impact of cancer upon Veterans’ lives over time. Interviewers reviewed the following with Veterans prior to conducting these interviews: general length of the interview (approximately 2 hours), scheduled breaks during the interview session (with the option for additional breaks as needed), options to complete the interview if the participant was unable to during their scheduled session, and an overview of the types of questions that will be asked (e.g. standardized scales). Individual measures for each interview protocol will be briefly described below.

**Table 2:** Overview of key interview measures

**Measures**	**Qualitative**	**Quantitative**	**T1: 6-month**	**T2: 12-month**	**T3: 18- month**
Adapted Illness Intrusive Ratings Scale (IIRS)		x	x		x
Alcohol Usage (AUDIT)		x			x
Benefit Finding Scale		x	x	x	x
Cancer Related Worry		x	x	x	x
Cancer-Related PTSD Symptoms (PCL-C)		x	x	x	x
Clinical Impressions (Interviewer Reflections)	x		x	x	x
Community Integration (PART)		x	x		x
Depression Assessment (PHQ 9)		x	x	x	x
Diagnosis and Treatment Experiences (Cancer Story)	x	x	x	x	x
Grip strength		x	x		x
Health literacy		x			x
Health values & goals	x	x		x	
Meaning making	x	x	x	x	x
Military Related PTSD (PC-PTSD)	x	x	x		
Montreal Cognitive Assessment (MoCA)		x	x		x
Previous Mental Health and Substance Abuse		x	x		
Quality of Life Assessment (PROMIS 29)		x	x	x	x
Sexual Functioning (Treatment Impact Scale)		x	x		x
Short Physical Performance Battery (SPPB)		x	x		x
Site Specific Quality of Life (EORTC QLQ)		x	x	x	x
Tobacco usage		x			x

### Time 1 interview (6 months)

The interview measures are presented in Table 2. The Time 1 (and 3) interview began with physical and cognitive performance screening measures. Veterans were asked, in general, if their memory, walking or balance, and body strength was worse, the same or better since their cancer diagnosis. Following these preliminary questions, interviewers performed the Montreal Cognitive Assessment (MoCA) [19] that assessed the Veterans’ visual constructional skills (e.g. ability to draw a picture), naming abilities (i.e. ability to name an object), memory, attention skills (e.g. ability to perform basic subtraction), sentence repetition, verbal fluency, abstraction (e.g. how two different objects are alike), delayed recall (e.g. repeating a list of words that were provided earlier in the interview) and orientation (e.g. knowing the current date and interview location). The Short Physical Performance Battery (SPPB) [20] assessed balance by having, for example, Veterans maintain standing poses for ten seconds with their feet placed in various positions (e.g. side-by-side and tandem) as well as strength by having Veterans attempt to stand up and sit down from a chair unassisted (e.g. repeated chair stand test). We also recorded Veterans gait speed as well as grip (i.e. hand) strength as part of our physical assessment measures.

Veterans’ background information was collected at Time 1, including demographic factors (age, race/ethnicity, education, and preferred language) as well as a brief history of substance abuse and mental health. Military-related, post-traumatic stress disorder (PTSD) was measured with the Primary Care PTSD (PC-PTSD) [21] screen that is used within the VHA to evaluate the presence or absence of combat-related PTSD. Open-ended questions were also asked of Veterans that further explored the relationship between cancer-related trauma and military post-traumatic stress.

Veterans’ illness narratives (i.e. “Cancer Story”) were elicited through a series of open-ended questions designed to document their diagnostic story. We asked participants “I would like to know the story of your diagnosis. What I mean is what caused you to be evaluated? How did you learn about the diagnosis?” Then we asked, “What was most stressful about the diagnostic experience?” Additional scripted prompts were used as necessary to elicit detailed information and responses were recorded verbatim. Additionally, a series of closed-ended questions based on the DSM-IV Criterion A1 and A2 PTSD [22] assessments followed this illness narrative and measured Veterans’ feelings about death, fear, helplessness and horror as well as the impact of cancer treatment upon the physical body. Types of treatments Veterans received (e.g. surgery, radiation, chemotherapy), their understanding of the cancer prognosis (e.g. active cancer, relapse, remission, cure, not sure, or other), and Veterans own and/or family history of cancer were also documented.

The emotional and physical effects of cancer were assessed through several measures. We chose to evaluate cancer-related PTSD symptoms using the PTSD Checklist-Civilian version (PCL-C) [23] to identify if at any time during the past four weeks Veterans may have experienced post-traumatic responses (e.g. repeated disturbing memories or dreams, avoidance, emotional numbness, or hyper-arousal) when thinking about their diagnostic and treatment experiences. Respondents were cued to consider specifically their cancer experiences in rating the PCL items. Additionally, the Cancer Related Worries Scale [24] evaluated Veterans’ level of cancer related worry in five domains: fear of recurrence, healthcare, family, existential, and recovery they may have experienced over the four weeks prior to this interview. To assess depression, we utilized the nine item Patient Health Questionnaire (PHQ-9) [25] that corresponds directly to the DSM criteria for major depression.

Quality of life was measured using the 29-item Patient Reported Outcomes Measurement Information System (PROMIS) [26] scale by examining Veterans’ physical function as well as levels of anxiety, depression, fatigue, sleep disturbance, sleep quality, ability to meet their social roles (e.g. work), and how much pain impacts their daily life and activities. An additional quality of life issue that arose several months after the study began involved many Veterans reporting their struggles with prophylactic dental extraction (e.g. in the case of oral cancers) and denture replacement. In response, we added supplementary questions to the interview protocol to more fully capture dental care concerns. Immediately following these questions, the European Organization for Research and Treatment of Cancer Quality of Life Questionnaire (EORTC QLQ) was included (questionnaires were tailored for the cancers included in this study: EORTC QLQ-CR29 [27] for colorectal, OE18 [28] for esophageal, and the H&N35 [29] for head and neck). These questionnaires evaluated the impact of cancer related symptoms (e.g. hair loss, pain, dry mouth, weight) experienced by Veterans in the week prior to their interview.

To address our interest in Veterans’ efforts to re-integrate back into their communities after cancer (i.e. re-engage with their social roles and activities held prior to cancer diagnosis), the Participation Assessment with Recombined Tools (PART) [6] questionnaire measured the level of importance particular roles and activities had in Veterans’ lives (e.g. school, work, housekeeping, relationships with family and friends, religion, recreation and leisure). Veterans rated these roles and activities from a level of low, medium or high life importance. Following these ratings, we asked Veterans to determine how much life satisfaction their medium to high level of importance activities had from a scale of 0 (total dissatisfaction) to 10 (high satisfaction). An additional measure, the adapted Illness Intrusive Ratings Scale (IIRS) [30], was utilized to measure the level of interference a cancer diagnosis brought to Veterans’ lives. Veterans were asked whether a series of pre-determined activities and feelings were hindered by the effects of their cancer (e.g. does cancer affect what I can eat, my hobbies, finances, religious participation, sexual life, relationships and self-improvement efforts). If cancer did cause interference with these activities and feelings, Veterans were then asked to rate this level of interference from 1 to 7, with 7 being the highest level of interference cancer brought to their lives.

We were also interested in measuring any positive outcome Veterans may have taken from their cancer experience and therefore employed the Benefit Finding Scale (BFS) [31] to evaluate post-traumatic growth. Veterans assessed how well their personality reflected a set of 22 characteristics (e.g. eating healthfully, exercise, concern for others, and coping well with stress), and whether they felt these characteristics had changed since experiencing cancer. For example, a participant may not have had a healthy diet prior to their cancer diagnosis, but improved their eating habits post-diagnosis. Given that sexual functioning is often impacted by cancer treatment, we utilized the Treatment Impact Scale [32] that measured whether Veterans’ physical abilities to perform and psychological interest in sex were affected by their cancer diagnosis and treatment, and to what level at Time 1 and 3. Veterans were reassured of their confidentiality in responding to these questions before interviewers administered this scale given the potential sensitive nature of this topic, and also had the option of completing these questions in private. Lastly, to address our interest in improving cancer survivorship health services for Veterans, we concluded the Time 1 interview with one final open ended question, “What one thing could the VA do better to support your cancer care needs?” to elicit ideas from Veterans on this topic.

### Time 2 interview (12 months)

The Time 2 interview began by asking Veterans for an update on their illness narratives with the open-ended question, “What is happening with your cancer now?” This question provided the Veterans an opportunity to report any updates on their treatment experiences since their previous interview. We followed this question by asking Veterans if they were currently in treatment, a date when their treatment ended (if it had), and reassessed cancer prognosis by inquiring how the Veterans conceptualized their current cancer status (e.g. active cancer, relapse, remission, cure, not sure and other). We also asked Veterans to reflect openly on what they found to be most stressful about their cancer treatment with the following question: “Looking back at your cancer treatment, what was most stressful?”

To address gaps in cancer health related services, Veterans were asked a series of closed-ended questions designed to measure their level of satisfaction with the VA health care system (e.g. medical care provided, education about their treatment, and social support). We also assessed various kinds of health care obstacles the Veterans may have faced while undergoing cancer treatment such as financial and/or transportation barriers, and social barriers such as fear and embarrassment. Veterans’ present and future healthcare needs were evaluated through the use of a pre-determined list of cancer care needs compiled by the American Society of Clinical Oncology (ASCO) (prevention and wellness, genetic risk, emotional or mental health, personal relationships, fertility, and financial advice or assistance). We shared this list with Veterans and asked them to respond with either a yes (I have a need for this), no (I do not have a need for this), or maybe to these various items. Veterans were then asked openly to provide any additional cancer survivorship needs not covered by the ASCO list.

To identify additional health care needs for cancer survivors, Veterans were provided a list of commonly used survivorship services (educational workshops, computer/Internet searches, online support groups, physical and occupational therapy, exercise, diet/supplements, journaling, meditation, yoga, massage, chiropractic care, acupuncture, psychotherapy, support groups, and anti-anxiety medication). We asked the Veterans to identify if they have ever used these services, or if they have not, if they cannot locate or are simply not interested in these particular services. To evaluate interest level among Veterans in using yoga to mediate some of their physical and psychological effects of cancer, we asked if they would come to a VA sponsored yoga class. If so, we asked Veterans to rate the likelihood they would find taking a yoga class embarrassing, whether they felt they were physically able to take yoga, and whether they felt learning yoga would benefit their overall health. These additional questions about yoga were added because of the reported health benefits of yoga for cancer survivors [33], in the context of a separate randomized trial of yoga being conducted.

To determine the impact of cancer on Veterans’ social lives and support systems, we asked Veterans if they have someone (e.g. spouse, confidant) they can turn to for emotional and instrumental support. We then had Veteran’s rate from a scale of 0 (not at all) to 4 (extremely) how their social relationships with family and/or friends have been impacted by their diagnosis (e.g. cancer straining these relationships, self-isolation from family/friends). These items were developed based on a pilot study [34].

At the Time 2 interview we were interested in Veterans’ abilities to make meaning from and to cope with cancer. Therefore we expanded our exploration of this realm by developing a series of questions based on the meaning making [35] and religion and spirituality in medical illness literatures [36-42]. We utilized a series of open-ended questions designed to elicit Veterans’ perceptions about the meaning of life and death since being diagnosed with cancer, as well as quantitative ratings about meaning making processes (see Additional file 2: Meaning Making: Time 2 Interview). We assessed the role of lifespan development [43] through the use of two open ended questions (“What qualities within yourself helped you most in coping with cancer?” and “Are there any qualities within yourself that made it harder to cope with cancer?”) as well as asking Veterans to rate if specific previous life challenges (e.g. combat, death of a loved one, divorce, addiction or alcoholism) helped them cope with their cancer. If Veterans identified some of these experiences as beneficial, we asked them to openly describe the ways these challenges helped them cope with their illness.

At Time 2 we developed the “Health Values and Goals” scale based on ours and others’ work [1,4,44]. This scale measured how much impact a cancer diagnosis and treatment had upon Veterans’ life goals and values. We asked Veterans to first review a pre-determined list of life values and/or goals that included, for example, taking care of myself (e.g. bathing, dressing), to walk or move around by myself, to avoid being a burden to others, and to make my own life decisions (e.g. about health, finances, housing). Veterans then chose from this list what they felt would be their most important life value or goal and assigned a rating of 10 (the highest rating meaning “I could not live without this one”). Veterans were then asked to choose the life value or goal of least importance and rate it numerically lower in importance to all the others in the list. The remaining goals and values were then rated between these two values; ratings could be repeated. Upon completion of this rating, Veterans were asked to reflect whether they achieved these values and goals since their cancer diagnosis and treatment, using a scale from 1 (I am not achieving this goal/value) to 5 (I am achieving this goal/value).

We concluded the Time 2 interview by asking Veterans to think about their futures and respond to a series of open-ended questions regarding this domain (“Now that you have had cancer, and may face ongoing decisions about medical care in the future, what would you want your family, friends, and/or doctors to know about you, in terms of what is most important to you in your life?” “If your cancer were to recur, is there anything you’d want to be sure your loved ones knew about you and your goals of care?”). To gather additional data on how to improve cancer care services for Veterans, we again asked Veterans to discuss what they felt the VA could improve upon in the provision of cancer care (“What one thing could the VA do better to support your cancer care needs?”).

In addition to these measures, we also re-administered the following scales from the Time 1 interview again at Time 2 to measure any functional, social and psychological changes in the past six months: BFS, PCL-C, VA CWADS V1, PHQ 9, PROMIS 29, and the EORTC QLQ. We omitted the cognitive and physical performance screening measures (MOCA, SPPB and grip strength) collected at Time 1 and chose to re-administer these measures again at Time 3 to avoid undue burden on the patient.

### Time 3 interview (18 months)

The Time 3 interview asked Veterans to provide an update on their illness narrative (“What is happening you’re your cancer now?”) to document any changes in their treatment experiences since the completion of their Time 2 interview 6 months prior. We also asked Veterans to reflect on how their life has changed for them since being diagnosed with cancer 18 months ago with the open-ended question, “From your perspective, what has changed over the 18 months since you were diagnosed?” We asked Veterans again whether they were receiving treatment at that time, the date their treatment ended, as well as how they conceptualized their prognosis (e.g. active, relapse, remission, cure, not sure and other). Additionally, this interview included a set of questions asking Veterans whether they had to make a medical decision to continue or re-start their cancer treatment after their initial treatment phase. If so, we asked what that medical decision was, and the factors involved in the Veterans’ decision-making process.

A tobacco assessment drawn from the Smoking History and the Fagerstrom Tolerance Questionnaires [45] was added to this interview. We asked Veterans if they currently smoked, and if so, to recall the number of cigarettes they smoked per day over the last seven days. Besides cigarettes, Veterans were also asked if they smoked cigars or pipes, or use smokeless tobacco. In addition to the tobacco assessment, alcohol usage was measured using the Alcohol Use Disorders Identification Test (AUDIT) which focused on the frequency of alcohol consumption (e.g. number of alcoholic beverages consumed on an average day). Additionally, we included Health Literacy Screening Questions [46] as part of this interview protocol to assess the level of difficulty Veterans may have had in understanding written medical information such as educational materials provided to patients and medical forms.

We also included a series of open-ended questions at Time 3 that asked Veterans to reflect upon their entire cancer experience from diagnosis to 18 months. Veterans described the most difficult aspect(s) of their illness experience (“In thinking about your whole cancer experience, what has been the hardest part of having cancer?”), what they believed will cause them the most struggle in the future (“In thinking about the future of dealing with this illness, what is the hardest thing for you to face?”), and what they believed helped them cope with their cancer (“What helped you the most in facing the diagnosis and treatment of, and recovery from cancer?”). We again asked Veterans for their thoughts and opinions on how the VA could improve cancer survivorship health services.

In addition to the measures described above, we re-assessed the following measures and scales from the Time 1 and 2 interviews: MoCA, SPPB, grip strength, PCL-C, VA CWADS V1, PHQ 9, Promise 29, EORTC QLQ, PART, adapted IIRS, BFS well as the Treatment Impact Scale.

### Analyses

The longitudinal study design allowed for continuous data analysis as study modules were completed and the completeness of data verified. Analyses include: quantitative analyses on item endorsement (yes/no) frequency, item mean and total scale score mean and range for cognition, physical capacity, cancer PTSD, worry, depression, quality of life, illness intrusiveness, and sexual function, and qualitative analyses of diagnostic and treatment experiences and meaning-making. Upon completion of our data collection, we will conduct quantitative analyses comparing our measures across the 6, 12 and 18 month time points to test our hypotheses. To determine the longitudinal course of functional disability and psychological distress in Veterans treated for cancer (specific aim 1) we will evaluate multivariate models in the form of multiple regressions. Path analysis will be constructed to adjust these assessments of longitudinal changes using groups of covariates (disease and treatment variables; demographic variables; social support; and pre-existing mental health variables). We will conduct similar multivariate analyses to determine the impact of disability and distress on community integration in Veterans treated for cancer, and the variables that mediate this relationship (specific aim 2).

To characterize gaps in Veterans Health Administration (VHA) rehabilitative services for Veterans following cancer (specific aim 3) we will calculate means and item frequencies for medical, emotional and educational care, and obstacles to receiving health care services. We will conduct mean and standard deviations to determine Veterans’ interests in support services such as mental health, CAM, rehabilitation and educational services. Additionally, we will calculate bivariate correlations comparing whether Veterans with mental health issues are interested in mental health care and if those with functional limitations will be interested in rehabilitative and CAM services. To characterize patient-centered goals for rehabilitation, community integration, and cancer survivorship treatment planning for Veterans following cancer (specific aim 4) we will calculate item frequencies for patient-centered goals. We will conduct qualitative analyses on the open-ended responses using both inductive and deductive coding methodologies developing at least an 80% inter-rater consistency rating. We will then compare the coded open-ended results with the closed ended ratings.

## Discussion

The VetCaReS project team is led by principal investigators with backgrounds in clinical psychology and internal medicine, both specialists in gerontology/geriatrics. Additional team members are drawn from diverse disciplines in the behavioral and social sciences (clinical and social psychology and anthropology) bringing a breadth of theoretical and analytical expertise to the study. Each week members of this multidisciplinary team participated in a teleconference between the Boston and Houston VA research sites to discuss a variety of operational issues associated with the study such as administrative tasks, recruitment, data collection, analyses, and dissemination of study results.

An administrative meeting agenda is circulated among team members, providing an overview of issues to discuss. Detailed recruitment reports, compiled and updated by the project coordinators, are discussed during these weekly calls. These reports note the interview module (Time 1, Time 2 and Time 3) and research site (Boston or Houston VAMCs), the number of expected interviews for a given month, followed by a detailed reporting of the number of Veterans contacted for an interview (those called and consenting to be interviewed, those called and refusing to be interviewed, and the number of Veterans mailed recruitment letters). The total number of interviews completed for each module and site, the number of Veterans remaining to be interviewed, the final number of overall valid interviews, and site totals were also included on these reports.

These weekly meetings also included detailed case study reports of recently completed interviews followed by a discussion of the case among team members. These case study reports provided a summary of the Veterans’ age, combat status, cancer type, stage, treatments, and overall physical and emotional health integrating responses across instruments and other factors that may complicate the Veterans case (e.g. stroke). Interviewers also provided their thoughts on what was “most interesting or significant about this interview,” if applicable, how the VA “could better help this Veteran,” and if applicable, the reasons the Veteran “has been able to cope.” The purpose of these case discussions was twofold. First, project principal (JM) and co-principal (AN) investigators could hear from interviewers on the technical aspects of the research process which might affect the validity of the data. Early on in the research process, we were able to identify what newly added or open-ended/non-standardized questions as well as response categories were working well, and those that required revision. Second, these discussions also provided study team members not directly involved in the data collection process the opportunity to be introduced to the Veterans and hear their stories. While interviewers were able to interact with the Veterans directly and develop rapport, the presentation of these case studies enabled other members of the study team to understand responses to standardized surveys that reflect the individual more fully. Many of these case study discussions brought to light relevant issues specific to this study population, pathways for future analyses, as well as dialogue on the more reflexive aspects of the research process itself. Highlights from these team discussions are discussed below:

• Stoma Reversal: We discussed the case of a veteran in his 60s diagnosed with Stage III colorectal cancer, and treated with surgery, which included a stoma, and chemotherapy. This patient experienced treatment related physical and emotional problems including pain, fatigue, weakness, eating and memory issues, as well as depression and difficulty sleeping. The patient’s ongoing struggle with his stoma has negatively impacted his quality of life, and his reversal has been delayed due to other necessary surgeries. We discussed the psychological impact for Veterans of a delayed stoma reversal, and discussed how the patient’s personal goals have been integrated into his post-surgical care.

• Teeth and Dentures: Some Veterans underwent prophylactic removal of all teeth in advance of radiation therapy. Our interviews revealed that not only did the loss of their teeth negatively affect their quality of life, but they faced numerous difficulties receiving dental care and, at-times, dentures. We felt additional questions were needed in our interview protocols to address this issue, and we also addressed the issue through consultation at local and national levels.

• Treatment Refusal: In the process of conducting this study, the issue regarding treatment refusal arose. There were a small number of Veterans (n = 6) who had their initial treatment, but refused further intervention, while two refused treatment altogether (n = 2). We discussed the possibility of creating separate variables to compare the Veterans who opted for all their treatment to those who refused some portions of treatment. Given the study’s purpose of capturing both diagnostic and treatment experiences, we reached consensus to remove the two “refusal” patients and place them in a separate database for future analysis. The discovery of this subset of Veterans in the data set led us to consider examining Veterans’ reasons behind their medical decision to refuse all or some portion of their treatment protocol.

• Challenges of the Interview Process: Weekly case discussions not only provided interviewers the opportunity to debrief the study team, but to discuss the personal impact of the interview process. These discussions revealed how developing a rapport with these Veterans, many whom interviewers had been interacting with for a year, caused mixed emotions. While it gave interviewers a good feeling to close their last interview seeing Veterans on the road to recovery, it was difficult to see those Veterans who were still struggling to regain their health 18 months after their initial diagnosis.

• Post-Traumatic Growth: Our case discussions revealed that not all Veterans conceptualized cancer as a negative life experience, but rather as an experience that brought post-traumatic growth. For example, a white male in his 50s diagnosed with Stage II colorectal cancer and treated with surgery described feeling “totally shocked” when he was diagnosed, and “was white as a ghost when they [the doctors] told me.” He experienced depression and “no support” from his ex-wife at this time. However, he has since changed his outlook on life. He reports being much happier, changed his diet to eat more healthfully, and is now a practicing Buddhist. He also reported disengaging from negative social relationships that he felt were pulling him down psychologically. Although he does fear recurrence, he does not let that fear overrule his life.

In addition to these case study reports, study team meetings included updates on current data checks. As each study module (Time 1, Time 2, and Time 3) was closed, the completeness of the data set was verified for each site. The study team was also updated on the progress of the Medical Record Review (MRR) conducted to verify the self-reported medical information provided by the Veterans. Pertinent IRB issues (e.g. amendments, yearly renewals) as well as funding issues (e.g. participant payments) were also reported during these meetings. Weekly meetings also facilitated ongoing discussions regarding the dissemination of research results. Results in the form of proposed and completed data analyses, conference presentations as well as publications were shared in this diverse, multidisciplinary environment where team members provided constructive feedback.

## Conclusions

There is currently a lack of cancer survivorship research focusing on aging Veterans. Therefore, the purpose of this study was to document the functional, psychosocial effects, and quality-of-life changes related to cancer diagnosis and treatment from time of diagnosis to 18 months for aging Veterans, and to identify the unique survivorship needs of this population. We anticipate that results from this study will support some of our hypotheses, and provide new knowledge to the wider literature on cancer survivorship by revealing a greater understanding of this experience from the perspective of aging Veterans as well as identify areas for future survivorship interventions. Our study is innovative given the scope and diversity of measures utilized within a longitudinal study design, allowing us to document any functional and psychosocial changes over time. Additionally, our weekly teleconferences illustrate the iterative nature of our research, allowing for refinement of study tools and highlighting important issues for discussion, analyses and further investigation. We anticipate that results from this study will be useful in designing intervention tools to improve the cancer survivorship experience for aging Veterans.

## Abbreviations

(PTSD): Post traumatic stress disorder; (IOM): Institute of Medicine; (VHA): Veterans Health Administration; (VA): Veterans Affairs; (IRB): Institutional Review Board; (CPRS): Computerized Patient Records System; (HIPAA): Health Insurance Portability and Accountability Act; (AJCC): American Joint Committee on Cancer; (MoCA): Montreal Cognitive Assessment; (SPPB): Short Physical Performance Battery; (PC-PTSD): Primary Care PTSD; (PCL-C): PTSD Checklist-Civilian version; (PHQ-9): Patient Health Questionnaire; (PROMIS): Patient Reported Outcomes Measurement Information System; (EORTC QLQ): European Organization for Research and Treatment of Cancer Quality of Life Questionnaires (Colorectal CR29, Esophageal OE18, Head and neck H&N35); (PART): Participation Assessment with Recombined Tools; (IIRS): Illness Intrusive Ratings Scale; (BFS): Benefit Finding Scale; (ASCO): American Society of Clinical Oncology; (AUDIT): Alcohol Use Disorders Identification Test; (MRR): Medical Record Review.

## Competing interests

The author(s) declare that they have no competing interests.

## Authors’ contributions

AN: Co-principal Investigator of the study protocol and grant proposal, Houston VA site leader, co-developed study design and methods, significant contribution to data analysis and dissemination of study results. LM: Wrote first draft of manuscript, contributed to data analysis and dissemination of study results. MK: Co-Investigator at Boston site; collaborated on developing study design and methods, contribution to data analysis and dissemination of study results. JSW: Assisted in the development of the study design and methods; interviewing. EM: Collaborator on the study, significant contribution in data analysis and dissemination of study results. JG: Study coordinator at Boston VA site, involved in all aspects of screening, recruitment, interviewing, data analysis and dissemination of study results. LH: Study coordinator at Houston VA site, involved in all aspects of screening, recruitment, interviewing, data analysis and dissemination of study results. JM: Principal investigator of the study protocol and grant proposal, Boston VA site leader, developed study design and methods, significant contribution to data analysis and dissemination of study results. All authors have read and approved the contents of this manuscript.

## Pre-publication history

The pre-publication history for this paper can be accessed here:

http://www.biomedcentral.com/1472-6963/13/93/prepub

## Supplementary Material

Additional file 1 **International Statistical Classification of Diseases and Related Health Problems (ICD-9) Codes for Cancers included in the study. **ICD-9 Codes utilized in the screening and recruitment process. Click here for file

Additional file 2 **Meaning Making: Time 2 Interview. **Questions intending to ask about how the individual makes sense of the cancer experience and copes with it. Click here for file

## References

[B1] Karel MJ Moye J Bank A Azar AR Three methods of assessing values for advance care planning J Aging Health 2007 19 1 123 151 10.1177/0898264306296394 17215205 PMC4859331

[B2] American Cancer Society Cancer Facts and Figures 2012 2012 Atlanta: American Cancer Society 500812

[B3] Siegel R DeSantis C Virgo K Stein KD Mariotto A Smith T Cooper D Gansler T Lerro C Fedewa S Cancer treatment and survivorship statistics, 2012 CA Cancer J Clin 2012 62 4 220 241 10.3322/caac.21149 22700443

[B4] Pearlman RA Starks H Cain KC Cole WG Improvements in advance care planning in the veterans affairs system: results of a multifaceted intervention Arch Intern Med 2005 165 6 667 674 10.1001/archinte.165.6.667 15795344

[B5] Institute of Medicine (IOM) From Cancer Patient to Cancer Survivor: Lost in Transition 2005 Washington, DC: The National Academic Press

[B6] Bogner JA Whiteneck GG Corrigan JD Lai JS Dijkers MP Heinemann AW Comparison of scoring methods for the participation assessment with recombined tools objective Arch Phys Med Rehabil 2011 92 4 552 563 10.1016/j.apmr.2010.11.014 21367397

[B7] Khan NF Rose PW Evans J Defining cancer survivorship: a more transparent approach is needed J Cancer Surviv 2012 6 1 33 36 10.1007/s11764-011-0194-6 21904942

[B8] Rowland JH Yancik R Cancer survivorship: the interface of aging, comorbidity, and quality care JNCI: J Nat Cancer Inst 2006 98 8 504 505 10.1093/jnci/djj154 16622113

[B9] Rao AV Demark-Wahnefried W The older cancer survivor Crit Rev Oncol Hematol 2006 60 2 131 143 10.1016/j.critrevonc.2006.06.003 16965920

[B10] Yancik R Wesley MN Ries LAG Havlik RJ Edwards BK Yates JW Effect of Age and comorbidity in postmenopausal breast cancer patients aged 55 years and older JAMA 2001 285 7 885 10.1001/jama.285.7.885 11180731

[B11] Hewitt M Rowland JH Yancik R Cancer survivors in the United States: Age, health, and disability J Gerontol A Biol Sci Med Sci 2003 58A 1 82 91 12560417 10.1093/gerona/58.1.m82

[B12] Bellury LM Ellington L Beck SL Stein KD Pett M Clark J Elderly cancer survivorship: An integrative review and conceptual framework Eur J Oncol Nurs 2011 15 233 242 10.1016/j.ejon.2011.03.008 21530396

[B13] Institute of Medicine (IOM) Cancer in Elderly People: Workshop Proceedings 2007 Washington, DC: The National Academic Press

[B14] Moye J Schuster JL Latini DM Naik AD The future of cancer survivorship care for veterans Fed Pract 2010 27 3 36 43 21318051 PMC3035919

[B15] Hilgeman M Moye J Archambault E Billings R Karel MJ Gosian J Naik A In the veterans voice Fed Pract 2012 29 Suppl 51S 59S PMC4364517 25798043

[B16] Jahn A Herman L Schuster J Naik A Moye J Distress and resilience after cancer in military veterans Res Hum Dev 2012 9 229 247 10.1080/15427609.2012.705555 PMC4358230 25774100

[B17] Moye J Schuster JL Latini DM Naik A The future of cancer survivorship care for veterans Fed Pract 2010 37 36 42 21318051 PMC3035919

[B18] Ganz PA The ‘three Ps’ of cancer survivorship care BMC Med 2011 9 1 14 16 10.1186/1741-7015-9-14 21310037 PMC3060853

[B19] Nasreddine ZS Phillips NA Bédirian V Charbonneau S Whitehead V Collin I Cummings JL Chertkow H The Montreal Cognitive Assessment (MoCA): a brief screening tool for mild cognitive impairment J Am Geriatr Soc 2005 53 695 699 10.1111/j.1532-5415.2005.53221.x 15817019

[B20] Freire AN Guerra RO Alvarado B Guralnik JM Zunzunegui MV Validity and reliability of the short physical performance battery in two diverse older adult populations in Quebec and Brazil J Aging Health 2012 24 5 863 878 10.1177/0898264312438551 22422762

[B21] Prins A Ouimette P Kimerling R Camerond RP Hugelshofer DS Shaw-Hegwer J Thrailkill A Gusman FD Sheikh JI The primary care PTSD screen (PC-PTSD): development and operating characteristics Prim Care Psychiatry 2004 9 1 9 14 10.1185/135525703125002360

[B22] American Psychiatric Association Diagnostic and statistical manual of mental disorders (Fourth edition text revision) DSM-IV-TR 2000 Washington, DC: American Psychiatric Association

[B23] Weathers FW Litz BT Herman DS Huska JA Keane TM The PTSD checklist (PCL): Reliability, validity, and diagnostic utility Annual Convention of the International Society for Traumatic Stress Studies 1993 San Antonio, TX: http://www.pdhealth.mil/library/downloads/pcl_sychometrics.doc

[B24] Moye J Schuster J Mulligan E Doherty K Naik A Assessing worry after cancer treatment 2012

[B25] Kroenke K Spitzer RL Williams JB The PHQ-9: validity of a brief depression severity measure J Gen Intern Med 2001 16 606 613 10.1046/j.1525-1497.2001.016009606.x 11556941 PMC1495268

[B26] PROMIS Health Organization and the PROMIS Cooperative Group Patient Reported Outcomes Measurement Information System (PROMIS) http://www.nihpromis.org

[B27] Gujral S Conroy T Fleissner C Sezer O King PM Avery KL Sylvester P Koller M Sprangers MG Blazeby JM Assessing quality of life in patients with colorectal cancer: An update of the EORTC quality of life questionnaire Eur J Cancer 2007 43 10 1564 1573 10.1016/j.ejca.2007.04.005 17521904

[B28] Blazeby JM Conroy T Hammerlid E Fayers P Sezer O Koller M Arraras J Bottomley A Vickery CW Etienne PL Clinical and psychometric validation of an EORTC questionnaire module, the EORTC QLQ-OES18, to assess quality of life in patients with oesophageal cancer Eur J Cancer 2003 39 10 1384 1394 10.1016/S0959-8049(03)00270-3 12826041

[B29] Osthus AA Aarstad AKH Olofsson J Aarstad HJ Head and neck specific Health Related Quality of Life scores predict subsequent survival in successfully treated head and neck cancer patients: a prospective cohort study Oral Oncol 2011 47 10 974 979 10.1016/j.oraloncology.2011.07.010 21856209

[B30] Devins G Binik Y Hutchinson T Hollomby D Barré P Guttmann R The emotional impact of End-stage renal disease: importance of Patients’ perceptions of intrusiveness and control Int J Psychiatry Med 1983 13 4 327 343 10.2190/5DCP-25BV-U1G9-9G7C 6671863

[B31] Tomich PL Helgeson VS Is finding something good in the bad always good? Benefit finding among women with breast cancer Health Psychol 2004 23 1 16 23 14756599 10.1037/0278-6133.23.1.16

[B32] Syrjala KL Schroeder TC Abrams JR Atkins TZ Brown WS Sanders JE Schubert MA Heiman JR Sexual function measurement and outcomes in cancer survivors and matched controls J Sex Res 2000 37 3 213 225 10.1080/00224490009552042

[B33] Sohl SJ Schnur JB Daly L Suslov K Montgomery GH Development of the beliefs about yoga scale Int J Yoga Ther 2011 21 85 91 PMC3360551 22398348

[B34] Moye J Jahn A Schuster J Gosian J Herman L Naik A Team tVR Distress and adaptations 6–12 months following oral-digestive cancer diagnoses in military veterans In proceedings for the Biennial Cancer Survivorship Research Conference 2012 Arlington, VA http://www.cancer.org/subsites/survivorship2012/index

[B35] Park CL Edmondson D Fenster JR Blank TO Meaning making and psychological adjustment following cancer: The mediating roles of growth, life meaning, and restored just-world beliefs J Consult Clin Psychol 2008 76 5 863 875 18837603 10.1037/a0013348

[B36] Trevino K Archambault E Schuster J Hilgeman M Moye J Religiosity and spirituality in military veteran cancer survivors Psychosoc Oncol 2011 29 619 635 10.1080/07347332.2011.615380 PMC4348693 22035535

[B37] Trevino K Archambault E Schuster J Richardson P Moye J Religious coping and psychological distress in military veteran cancer survivors J Relig Health 2012 51 1 87 98 10.1007/s10943-011-9526-0 21822744 PMC4859334

[B38] Marler PL Hadaway CK “Being Religious” or “Being Spiritual” in America: A Zero-sum proposition? J Sci Study Relig 2002 41 2 289 300 10.1111/1468-5906.00117

[B39] Hebert R Zdaniuk B Schulz R Scheier M Positive and negative religious coping and well-being in women with breast cancer J Palliat Med 2009 12 6 537 545 10.1089/jpm.2008.0250 19508140 PMC2789454

[B40] Sherman AC Plante TG Simonton S Latif U Anaissie EJ Prospective study of religious coping among patients undergoing autologous stem cell transplantation J Behav Med 2009 32 1 118 128 10.1007/s10865-008-9179-y 18855130

[B41] Balboni TM Paulk ME Phelps A Wright A Peteet J Block S Lathan C VanderWeele T Prigerson H Support of cancer patients’ spiritual needs and associations with medical care costs at the end of life Cancer (0008543X) 2011 117 23 5383 5391 10.1002/cncr.26221 PMC3177963 21563177

[B42] Pargament KI Smith BW Koenig HG Perez L Patterns of positive and negative religious coping with major life stressors J Sci Study Relig 1998 37 4 710 724 10.2307/1388152

[B43] Blank TO Bellizzi KM A gerontologic perspective on cancer and aging Cancer 2008 112 11 2569 2576 10.1002/cncr.23444 18428204

[B44] Schulman-Green DJ Naik AD Bradley EH McCorkle R Bogardus ST Goal setting as a shared decision making strategy among clinicians and their older patients Patient Educ Couns 2006 63 1/2 145 151 16406471 10.1016/j.pec.2005.09.010 PMC10791156

[B45] Pomerleau CS Carton SM Lutzke ML Flessland KA Pomerleau OF Reliability of the fagerstrom tolerance questionnaire and the fagerstrom test for nicotine dependence Addict Behav 1994 19 1 33 39 10.1016/0306-4603(94)90049-3 8197891

[B46] Chew LD Bradley KA Boyko EJ Brief questions to identify patients with inadequate health literacy Fam Med 2004 36 8 588 15343421

