# Supplementary material for: Cancer survivor rehabilitation and recovery: Protocol for the Veterans Cancer Rehabilitation Study (Vet-CaRes)
Source: BMC Health Serv Res. 2013 Mar 11;13:93. doi: 10.1186/1472-6963-13-93 (PMC3626766; doi:10.1186/1472-6963-13-93)
Supplement: Additional file 1 — International Statistical Classification of Diseases and Related Health Problems (ICD-9) Codes for Cancers included in the study. ICD-9 Codes utilized in the screening and recruitment process. [file 1472-6963-13-93-S1.docx]

Additional File 1:

International Statistical Classification of Diseases and Related Health Problems (ICD-9) Codes for Cancers included in the study

Head and Neck (“Oral”) Cancers – ENT doctors

140.0 MAL NEO UPPER VERMILION

140.1 MAL NEO LOWER VERMILION

140.3 MAL NEO UPPER LIP, INNER

140.4 MAL NEO LOWER LIP, INNER

140.5 MAL NEO LIP, INNER NOS

140.6 MAL NEO LIP, COMMISSURE

140.8 MAL NEO LIP NEC

140.9 MAL NEO LIP/VERMIL NOS

141.0 MAL NEO TONGUE BASE

141.1 MAL NEO DORSAL TONGUE

141.2 MAL NEO TIP/LAT TONGUE

141.3 MAL NEO VENTRAL TONGUE

141.4 MAL NEO ANT 2/3 TONGUE

141.5 MAL NEO TONGUE JUNCTION

141.6 MAL NEO LINGUAL TONSIL

141.8 MALIG NEO TONGUE NEC

141.9 MALIG NEO TONGUE NOS

142.0 MALIG NEO PAROTID

142.1 MALIG NEO SUBMANDIBULAR

142.2 MALIG NEO SUBLINGUAL

142.8 MAL NEO MAJ SALIVARY NEC

142.9 MAL NEO SALIVARY NOS

143.0 MALIG NEO UPPER GUM

143.1 MALIG NEO LOWER GUM

143.8 MALIG NEO GUM NEC

143.9 MALIG NEO GUM NOS

144.0 MAL NEO ANT FLOOR MOUTH

144.1 MAL NEO LAT FLOOR MOUTH

144.8 MAL NEO MOUTH FLOOR NEC

144.9 MAL NEO MOUTH FLOOR NOS

145.0 MAL NEO CHEEK MUCOSA

145.1 MAL NEO MOUTH VESTIBULE

145.2 MALIG NEO HARD PALATE

145.3 MALIG NEO SOFT PALATE

145.4 MALIGNANT NEOPLASM UVULA

145.5 MALIGNANT NEO PALATE NOS

145.6 MALIG NEO RETROMOLAR

145.8 MALIG NEOPLASM MOUTH NEC

145.9 MALIG NEOPLASM MOUTH NOS

146.0 MALIGNANT NEOPL TONSIL

146.1 MAL NEO TONSILLAR FOSSA

146.2 MAL NEO TONSIL PILLARS

146.3 MALIGN NEOPL VALLECULA

146.4 MAL NEO ANT EPIGLOTTIS

146.5 MAL NEO EPIGLOTTIS JUNCT

146.6 MAL NEO LAT OROPHARYNX

146.7 MAL NEO POST OROPHARYNX

146.8 MAL NEO OROPHARYNX NEC

146.9 MALIG NEO OROPHARYNX NOS

147.0 MAL NEO SUPER NASOPHARYN

147.1 MAL NEO POST NASOPHARYNX

147.2 MAL NEO LAT NASOPHARYNX

147.3 MAL NEO ANT NASOPHARYNX

147.8 MAL NEO NASOPHARYNX NEC

147.9 MAL NEO NASOPHARYNX NOS

148.0 MAL NEO POSTCRICOID

148.1 MAL NEO PYRIFORM SINUS

148.2 MAL NEO ARYEPIGLOTT FOLD

148.3 MAL NEO POST HYPOPHARYNX

148.8 MAL NEO HYPOPHARYNX NEC

148.9 MAL NEO HYPOPHARYNX NOS

149.0 MAL NEO PHARYNX NOS

149.1 MAL NEO WALDEYER'S RING

149.8 MAL NEO ORAL/PHARYNX NEC

149.9 MAL NEO OROPHRYN ILL-DEF

Esophageal Cancers

150.0 MAL NEO CERVICAL ESOPHAG

150.1 MAL NEO THORACIC ESOPHAG

150.2 MAL NEO ABDOMIN ESOPHAG

150.3 MAL NEO UPPER 3RD ESOPH

150.4 MAL NEO MIDDLE 3RD ESOPH

150.5 MAL NEO LOWER 3RD ESOPH

150.8 MAL NEO ESOPHAGUS NEC

150.9 MAL NEO ESOPHAGUS NOS

Gastric Cancers

151.0 MAL NEO STOMACH CARDIA

151.1 MALIGNANT NEO PYLORUS

151.2 MAL NEO PYLORIC ANTRUM

151.3 MAL NEO STOMACH FUNDUS

151.4 MAL NEO STOMACH BODY

151.5 MAL NEO STOM LESSER CURV

151.6 MAL NEO STOM GREAT CURV

151.8 MALIG NEOPL STOMACH NEC

151.9 MALIG NEOPL STOMACH NOS

Small Bowl Cancers

152.0 MALIGNANT NEOPL DUODENUM

152.1 MALIGNANT NEOPL JEJUNUM

152.2 MALIGNANT NEOPLASM ILEUM

152.3 MAL NEO MECKEL'S DIVERT

152.8 MAL NEO SMALL BOWEL NEC

152.9 MAL NEO SMALL BOWEL NOS

Colorectal Cancers

153.0 MAL NEO HEPATIC FLEXURE

153.1 MAL NEO TRANSVERSE COLON

153.2 MAL NEO DESCEND COLON

153.3 MAL NEO SIGMOID COLON

153.4 MALIGNANT NEOPLASM CECUM

153.5 MALIGNANT NEO APPENDIX

153.6 MALIG NEO ASCEND COLON

153.7 MAL NEO SPLENIC FLEXURE

153.8 MALIGNANT NEO COLON NEC

153.9 MALIGNANT NEO COLON NOS

154.0 MAL NEO RECTOSIGMOID JCT

154.1 MALIGNANT NEOPL RECTUM

154.2 MALIG NEOPL ANAL CANAL

154.3 MALIGNANT NEO ANUS NOS

154.8 MAL NEO RECTUM/ANUS NEC

159.0 MALIG NEO INTESTINE NOS

Other Head and Neck to Include

160.0 MAL NEO NASAL CAVITIES

160.1 MALIG NEO MIDDLE EAR

160.2 MAL NEO MAXILLARY SINUS

160.3 MAL NEO ETHMOIDAL SINUS

160.4 MALIG NEO FRONTAL SINUS

160.5 MAL NEO SPHENOID SINUS

160.8 MAL NEO ACCESS SINUS NEC

160.9 MAL NEO ACCESS SINUS NOS

161.0 MALIGNANT NEO GLOTTIS

161.1 MALIG NEO SUPRAGLOTTIS

161.2 MALIG NEO SUBGLOTTIS

161.3 MAL NEO CARTILAGE LARYNX

161.8 MALIGNANT NEO LARYNX NEC

161.9 MALIGNANT NEO LARYNX NOS

Other Esophageal/Gastric to Include

238.1 MAL NEO CONNECTIVE/SOFT TISSUE
