# Supplementary material for: Cancer survivor rehabilitation and recovery: Protocol for the Veterans Cancer Rehabilitation Study (Vet-CaRes)
Source: BMC Health Serv Res. 2013 Mar 11;13:93. doi: 10.1186/1472-6963-13-93 (PMC3626766; doi:10.1186/1472-6963-13-93)
Supplement: Additional file 2 — Meaning Making: Time 2 Interview. Questions intending to ask about how the individual makes sense of the cancer experience and copes with it. [file 1472-6963-13-93-S2.docx]

Additional File 2:

MEANING MAKING: Time 2 Interview

Questions intending to ask about how the individual makes sense of the cancer experience and copes with it. Although meaning making and coping can be different concepts.

| 1. These next questions are about ways you may have coped or changed with cancer. In thinking of your life so far, what previous experiences, good and bad, have helped you prepare for or cope with cancer? How? |
| --- |
| 1. Have you ever tried to understand why you got cancer? Yes No   2A. *[Meaning of Cancer]* Why do you think you got cancer? |
| 2B. Now I would like you to think about that question more philosophically. Have you ever searched for the meaning, significance, or purpose of cancer in your life? Please describe: |
| 2C. *[Process]* If yes, How did you come to that understanding. What brought you to think this way? |
| 2D. If no, tell me more about why not. Why is this something you do not think about? |
| 1. How often do you ponder or try to understand why you were diagnosed with cancer?   Never  Rarely  Sometimes  Often  Very Often   1. How satisfied or dissatisfied do you feel you are with your understanding of why you got cancer?   Very Settled  Settled/Uncertain  Neutral  Unsettled/Uncertain  Very Unsettled   1. Imagine explaining to someone close to you the impact of cancer on your life, what would you say? |
| 1. Have close friends or family influenced the way you think about or understand cancer? |
| 1. 7A. Has cancer changed your view of life, or its meaning for you? Yes No   7B. *[Meaning of Life]* What is your view of life now? |
| 7C. *[Process]* How did having cancer cause you to change your view of life? |
| 1. 8A. *[Meaning of Death]* Has cancer changed your view of death, or its meaning for you? Yes No   8B. What is your view death now? |
| 8C. *[Process]* How did having cancer cause you to change your view of death? |
| 1. Have your religious or spiritual beliefs changed as a result of your cancer?   *If yes* How? More religious/spiritual Less religious/spiritual Other: |
| 1. Have your religious and spiritual beliefs made it difficult for you cope with cancer?   *If yes* How? |
| 1. Have you found yourself questioning, challenging, or turning away from your religious or spiritual beliefs as a result of your cancer?   *If yes* How? |
| 1. What qualities within yourself helped you most in coping with cancer |
| 1. Are there any qualities within yourself that made it harder to cope with cancer? |
| 1. (After asking if any life experiences helped in coping with cancer) …How did these experiences help you to cope with cancer? |
